# Cerebral Venous Sinus Thrombosis Following an mRNA COVID-19 Vaccination and Recent Oral Contraceptive Use

**DOI:** 10.3390/life13020464

**Published:** 2023-02-07

**Authors:** Timothy C. Frommeyer, Tongfan Wu, Michael M. Gilbert, Garrett V. Brittain, Stephen P. Fuqua

**Affiliations:** 1 Department of Pharmacology & Toxicology, Boonshoft School of Medicine, Wright State University, Dayton, OH 45435, USA; 2 Department of Neurology, Boonshoft School of Medicine, Wright State University, Dayton, OH 45435, USA

**Keywords:** cerebral venous thrombosis, COVID-19, vaccination, oral contraception, mRNA

## Abstract

Rising concerns of cerebral venous sinus thrombosis (CVST) and other forms of venous thromboembolism have been associated with the SARS-CoV-2 vaccinations. Adverse effects with vector-based vaccines are well documented in the literature, while less is known about the mRNA vaccines. This report documents a case of CVST in a 32-year-old female patient who received her second Pfizer mRNA COVID-19 vaccination 16 days prior to hospital admission and had started oral combined contraceptives approximately 4 months beforehand. Clinicians should be cognizant of the possibility that mRNA vaccines, when combined with other risk factors like oral contraceptive pill use, may enhance one’s hypercoagulable status.

## 1. Introduction

Cerebral venous sinus thrombosis (CVST) is a rare and potentially fatal cause of stroke. It can present with a multitude of signs and symptoms, which make it difficult to differentiate from other neurological conditions. As such, it is frequently overlooked due to the vague nature of its clinical and radiological presentation, making CVST a challenging diagnosis. Early recognition of its presentation and associated symptoms with prompt treatment can improve overall health outcomes and prognosis in patients [1].

CVST accounts for 0.5–1% of all strokes and is estimated to occur more often in females, with a 3:1 female-to-male ratio [2,3]. This stroke subtype is a multifactorial disease with 85% of affected adults having at least one risk factor [4]. While the pathophysiology is not fully understood, the main risk factors include prothrombotic conditions such as factor V Leiden, antiphospholipid syndrome, protein C deficiency, and antithrombin III deficiency [1,5]. Additional risk factors include infections, mechanical trauma, vasculitis, intracranial defects, hematological conditions, systemic diseases, and medications such as oral contraceptives pills (OCPs) [1]. 

Combined OCPs containing ethinylestradiol and progestogen are associated with an increased risk of venous thromboembolism (VTE) in women of reproductive age [6,7]. This relationship is related to OCP-induced changes in coagulation and fibrinolysis, which alters the hemostatic balance towards a prothrombotic direction [8,9]. In fact, the use of OCPs has been shown to increase the odds of CVST by 5- to 22-fold [10]. Another known risk factor for VTE that has recently emerged is infection with the novel coronavirus, SARS-CoV-2 [11]. In patients with combined OCPs, infection with COVID-19 is thought to aggravate the risk of VTE and strokes [12]. While the VTE risks of OCPs and COVID-19 are well-known, less is known about the thromboembolic effects of mRNA COVID-19 vaccines, especially in combination with OCPs. 

While COVID-19 vaccination administrations are being widely conducted to overcome the pandemic, an emerging concern about thromboembolic side effects has been realized. A case series of CVST and thrombocytopenia associated with virus vector COVID-19 vaccines (Johnson & Johnson (New Brunswick, NJ, USA); AstraZeneca (Cambridge, UK)) has been reported [13,14,15,16,17]. This vaccine associated syndrome, called vaccine-induced immune thrombocytopenia (VITT), has a pathogenesis similar to heparin-induced thrombocytopenia via immune-mediated platelet activation. Now widely recognized, the emergence of VITT resulted in regulatory actions by the Center for Disease Control and Prevention (CDC) and the Food and Drug Administration (FDA) in 2021.

Conversely, the thromboembolic side effects of mRNA COVID-19 vaccines (Pfizer (New York, NY, USA); Moderna (Cambridge, MA, USA)) have been rarely reported, with only a few case reports published within medical literature [18,19,20,21,22,23]. The underlying pathogenesis and clinical characteristic of VTE after mRNA COVID-19 vaccines has not been well explored when compared with VITT induced by virus vector COVID-19 vaccines. In addition, the relationship between VTE, COVID-19 vaccination, and OCP use in women of reproductive age is not well understood. Therefore, we have reported a rare case of CVST in a female on OCPs after an mRNA Pfizer COVID vaccination.

## 2. Case

A 32-year-old female with a history of depression and acne was presented to the Emergency Department (ED) with concern for stroke. She received her second Pfizer mRNA COVID-19 vaccination 16 days prior to admission and started oral combined contraceptives (OCPs) approximately 4 months prior. The patient does not have a personal or family history of atypical headaches, hypercoagulation disorders/thrombosis or autoimmune disease. She does not use tobacco products. Medications prior to admission included norgestimate-ethinyl estradioL (TRINESSA, 28,) 0.18/0.215/0.25 mg-35 mcg (28) Tablet, Sulfacetamide Sodium 10 % Cleanser, and Dapsone (ACZONE) 5 % topical gel. The patient was brought to the ED by her mother when she fell at home and was unable to talk or ambulate. She presented with aphasia, right hemiplegia, and collapse, subsequently resulting in a stroke alert upon arrival. Three days prior to presentation, the patient began to develop new onset headaches with no other associated symptoms. She was seen at an urgent care twice, where she was diagnosed with a migraine and prescribed sumatriptan and ibuprofen. The patient opted to stay with her parents due to the increasing severity of headaches.

Upon arrival to the ED, the patient’s vitals were blood pressure of 114/80 mmHg with a MAP of 93 mmHg, P 78 bpm, oxygen saturation of 100% on room air, axillary temperature of 96.9 °F (36.1 °C) and weight of 67.1 kg. The patient was awake and alert but unable to answer orientation questions secondary to expressive aphasia. She was able to follow most commands, however, she struggled with complex commands. Neurological physical exam findings were significant for expressive aphasia, left superior hemianopsia, right facial droop, ⅗ left shoulder strength (Motor strength scale 1 to 5: 0 = no muscle contraction; 1 = flicker or trace of contraction is seen; 2 = active movement in the same plane, not against gravity; 3 = active movement against gravity but not resistance; 4 = active movement against gravity with some resistance; 5 = active movement against gravity with full resistance), ⅕ right shoulder strength, flaccid paralysis of her right upper extremity and bilateral lower extremities. Deep tendon reflexes were all 2/4 except for 3 s at the right brachioradialis and bilateral knees. Upgoing Babinski reflex was noted on the right side but not the left side. Sensation was decreased on the right upper extremity compared with the left; bilateral lower extremities sensations were intact. 

The patient proceeded with the stroke workup. CT of the head showed relative hyper density of the superior sagittal, left transverse, and straight sinuses suggesting dural venous sinus thrombosis. No evidence of parenchymal or extra-axial hemorrhage were noted. CT angiogram of the head and neck revealed occlusion of the superior sagittal sinus, left transverse and sigmoid sinuses extending into the internal jugular vein, straight sinus, left internal cerebral vein. Nonocclusive thrombus involves the vein of Galen; right transverse sinus with normal contrast opacification of the right sigmoid sinus. There was no evidence of arterial stenosis, occlusion, or aneurysm in the head. Bilateral carotid systems and vertebral arteries were all enhanced normally. Extravascular findings were unremarkable. Her initial NIH Stroke Score (NIHSS) was 19. In the absence of other inciting injuries or underlying medical comorbidities, cerebral venous sinus thrombosis secondary to contraceptive use, COVID vaccinations, underlying autoimmune process, and occult malignancy was suspected. The patient was started with a high-dose heparin drip to prevent thrombus extension and inpatient seizure precautions with close neurologic monitoring. The patient was then transferred to a larger hospital for further workup and neurocritical management. 

**Figure 1:**
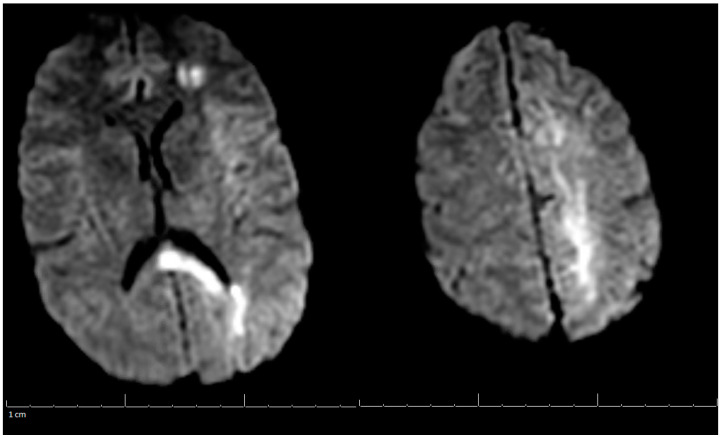
MRI of the Brain without contrast (DWI) (Magnetic Resonance Imaging (MRI) is a technique that uses magnetic fields and computer generated radio waves to create images of organs and tissues in your body. Magnetic Resonance Venography (MRV) uses magnetic resonance technology and intravenous contrast dye to visualize the veins. Diffusion-Weighted Imaging (DWI) is a form of MRI that uses the diffusion of water molecules to generate images).

**Figure 2:**
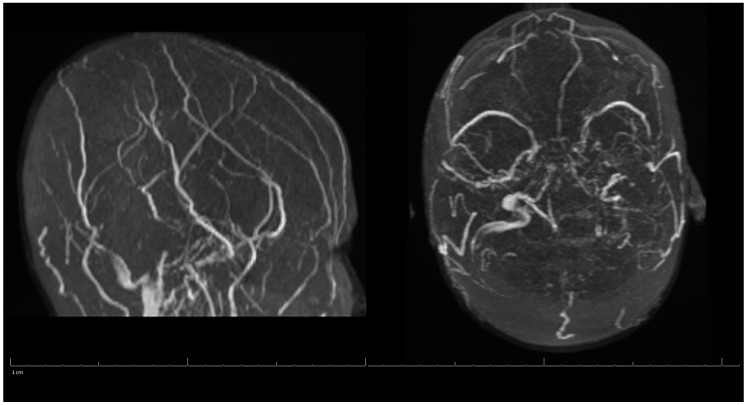
MRV of the Head during Hospital Admission.

Additional labs showed Complete Blood Count (CBC) within acceptable limits, Basic Metabolic Panel (BMP) significant with glucose of 142 mg/dL, normal cardiac troponin level, and a negative urine drug screen. Urinalysis showed a small amount of blood but no evidence of infection. Genetic hypercoagulable labs, including antinuclear antibody, antineutrophil cytoplasmic antibodies, anti-cardiolipin, anti-Beta-2-glycoprotein, Factor V Leiden, and a lupus anticoagulant assay, were also unremarkable. Several hypercoagulable tests including antithrombin, protein C, and protein S were deferred, as results would have been confounded by the acute thrombotic event. Of note, the patient had abnormal findings on the lipid panel consisting of high cholesterol, triglyceride, and VLDL, and a low HDL level. Ultrasound Venous Doppler of the bilateral lower extremities showed no deep vein thrombosis. MRI brain/MRV (Magnetic Resonance Imaging (MRI) is a technique that uses magnetic fields and computer-generated radio waves to create images of organs and tissues in your body. Magnetic Resonance Venography (MRV) uses magnetic resonance technology and intravenous contrast dye to visualize the veins. Diffusion-Weighted Imaging (DWI) is a form of MRI that uses the diffusion of water molecules to generate images) with and without contrast confirmed acute venous infarct in the left subcortical frontal and parietal lobes as well as the left splenium of the corpus callosum. Thrombosis of the superior sagittal sinus, cortical veins, straight sinus, left internal cerebral vein, vein of Galen and the bilateral transverse sinuses, as well as the left sigmoid sinus, were also confirmed. A small hemorrhagic focus in the left occipital lobe was noted and was likely venous hemorrhages (Figure 1 and Figure 2). The final diagnosis was diffuse cerebral venous sinus thrombosis likely related to OCP use with possible contribution by recent COVID vaccination. 

On hospital day two, the patient remained more aphasic than would be expected by her imaging results. A 24-h video electroencephalogram (EEG) was ordered to rule out nonconvulsive seizure activity secondary to CVST. The EEG did not show seizure activity but did show a high burden of abnormal epileptiform discharges, and levetiracetam (Keppra) 1000 mg twice daily was started for seizure prophylaxis. By hospital day three, the patient has significantly improved in motor strength, speech, and activity. NIHSS at this time was three. She was discontinued on the heparin drip and started on rivaroxaban (Xarelto), for long-term thrombus stabilization and prophylaxis against future clotting events, and atorvastatin (Lipitor) 40 mg. Her peripherally inserted central catheter (PICC) line, foley catheter, and intravenous fluids were also discontinued and she was transferred to a stepdown unit. She continued to improve clinically and was discharged on hospital day six to acute rehabilitation remaining on current medications. 

**Figure 3:**
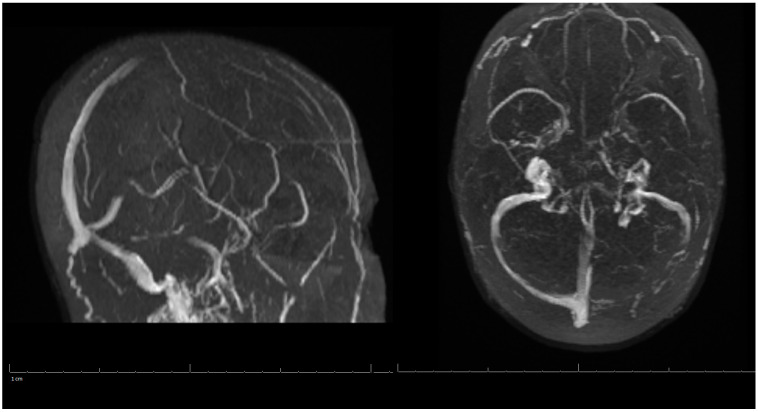
MRV of the Head on 3-Months Post-Hospitalization.

On her outpatient neurology clinic follow-up six weeks later, the patient was doing very well and largely back to her baseline. She still endorsed mild headaches about every other day and was using acetaminophen (Tylenol) for pain relief. The patient had been progressing well from attending regular physical therapy, occupational therapy, and speech therapy. Due to no seizure-like events, Keppra was to be weaned off to 500 mg every two weeks. Decreased Tylenol use was also suggested to avoid a rebound phenomenon. She was medically cleared to return to work and driving. Her three-month post-hospitalization MRV of the head showed stable left occipital intraparenchymal hemorrhage concerns for venous hemorrhage; partial thrombosis of the left transverse sinus improved in appearance compared with the previous examination three months ago; unremarkable appearance to the superior sagittal sinus, internal cerebral veins, inferior sagittal sinus, right transverse sinus, right and left sigmoid sinuses and internal jugular veins (Figure 3). A lipid panel showed improvement from inpatient levels. At the following telemedicine visit five months later, the patient reported only residual right hand weakness associated with reduced hand grip, but no further headaches. She discontinued Keppra at her last visit and has tolerated it well. Based on her most recent MRV, lipid panel, and clinical improvement, Xarelto and Lipitor were discontinued. The patient was advised to continue to follow a healthy diet and exercise to keep cholesterol under control, and avoid estrogen-containing birth control. There are no limitations on her activities from a neurological perspective. Another follow-up was scheduled in six months to ensure there are no new concerns, however, the patient did not show for this visit. 

## 3. Discussion

According to our literature review, this is the first published case report of CVST following recent completion of the Pfizer mRNA COVID-19 vaccination series alongside oral combined contraceptives in a reproductive age female in the United States. Dias and colleagues also reported a similar phenomenon in Portugal in a 47 year old female, though the length of time the patient had been on OCPs is unclear [19]. Globally, it appears the reporting rate of unexpected cerebral venous thrombosis for post COVID-19 mRNA vaccinations is 0.9% for mRNA-1273 (Moderna) and 0.4% for BNT1626b2 mRNA (Pfizer) [20]. Of note, this data may be challenging to assess due to differing databases. Park and colleagues analyzed the pharmacovigilance database from the World Health Organization and found 756 (0.07%) cases of cerebral venous thrombosis events out of 1,154,023 sampled mRNA COVID19 vaccines (620 (0.05% for Moderna, and 136 (0.01%) for Pfizer) [24]. In Singapore, as of 31 May 2021, 4,047,651 million doses of the mRNA vaccine had been delivered with three possible COVID-19 vaccine associated cerebral venous thrombosis events [20]. Finally, one recent systematic review found 11 cases of CVST that occurred following mRNA vaccinations [25]. Thus, as the data continues to evolve, the importance of quick recognition of cerebral venous thrombosis events is critical for patient outcomes. 

The pathophysiology of mRNA vaccine-induced VTE remains unclear, but some have proposed it may be due to endothelial dysfunction [20,21]. More specifically, the disruption of the blood-brain barrier may allow the spike glycoprotein to directly cause platelet aggregation, promote IL-6 inflammatory response, and activation of the alternative complement pathway [20,21]. This is in contrast to the proposed VITT mediated reaction which is due to PF4-reactive antibodies [16]. The mechanism behind endothelial dysregulation supports why OCPs, and other risk factors including hematologic disease or autoimmune disease, may increase the risk of a hypercoagulable state post-COVID19 mRNA vaccination. More specifically, OCPs affect blood clotting by increasing plasma fibrinogen, the activity of coagulation factors, and platelet activity while decreasing antithrombin III [26]. Thus, when combined with mRNA vaccines, a hypercoagulable state could potentially lead to obstruction of the cerebral sinuses when a thrombus does not resolve. It is important to consider that the COVID-19 infection has also been demonstrated to cause a hypercoagulable state and is responsible for a number of CVST’s [27,28,29]. The risk of a COVID-19 infection itself may be more likely to cause CVST in comparison to the mRNA vaccines [30]. More specifically, Taquet and colleges found the incidence of cerebral venous thrombosis two weeks after a COVID-19 diagnosis to be 42.8 million people (95% CI 28.5–64.2), which was significantly higher than in a matched cohort of people who received an mRNA vaccine (RR = 6.33, 95% CI 1.87–21.40, *p* = 0.00014) [30]. Another study analyzed over 200 different hospitals and found the incidence of CVST in COVID-19 hospitalized patients to be 231 per 100,000 person years (95% CI, 152.1–350.8) [31]. The mechanism appears to be similarly mediated through endothelial damage caused by viral protein binding to the ACE-2 receptor, resulting in endothelial damage promoting a hypercoagulable state [27]. 

It is very possible that this patient’s stroke was provoked by her recent COVID-19 vaccine, however, it is difficult to definitively prove. While we believe that the risk of coagulopathy provoked by mRNA vaccines should be further investigated, we do recognize that this presentation could be coincidental and not directly associated with the mRNA vaccination. CVST accounts for roughly 0.5–0.7% of all strokes and is commonly seen in younger populations [32]. It is more common in females than males, and a multitude of risk factors exist including genetic thrombophilia, infectious etiologies, and trauma [33]. In our patient, the CVST could have been due to OCPs alone, or there may be an underlying hematologic disorder that was not identified upon initial testing. Therefore, the patient would benefit from seeing a hematologist for further workups of genetic hypercoagulable conditions, including antithrombin, protein C, protein S, heparin induced thrombocytopenia antibodies, and specific coagulation factor activity levels.

In sum, the literature on COVID-19 vaccination and COVID-19 infections is challenging to report. Large data sets and the continually evolving COVID-19 pandemic make it difficult to draw specific conclusions. However, it is important for clinicians to consider that COVID-19 or mRNA vaccinations may predispose patients with risk factors for hypercoagulable states to CVSTs. 

## 4. Conclusions

This case report shows one rare instance of CVST in a patient who received her second dose of the mRNA vaccination and was recently put on OCPs. This is not to deter the usage of mRNA vaccination as demonstrated by an increased risk for CVST for patients who are infected with COVID-19. However, other risk factors, such as OCPs that result in a hypercoagulable state, may increase the risk for CVST in patients who receive the mRNA vaccination. Clinicians should consider COVID-19 vaccine-induced CVST in patients recently vaccinated. Early diagnosis and treatment may ultimately improve health outcomes.

## Data Availability

Not applicable.
